# Dr. Walter E. Gregory

**Published:** 1918-12

**Authors:** 


**Dr. Walter E. Gregory,** Buffalo 1889, long a member of the staff of the Jackson Health Resort, died at Dansville Oct. 26, of Pneumonia, complicating influenza. He had retired from practice but had volunteered for work during the epidemic.

